# Antiviral Potential of Small Molecules Cordycepin, Thymoquinone, and N6, N6-Dimethyladenosine Targeting SARS-CoV-2 Entry Protein ADAM17

**DOI:** 10.3390/molecules27249044

**Published:** 2022-12-19

**Authors:** Jiayue He, Shuguang Liu, Qi Tan, Zhiying Liu, Jiewen Fu, Ting Li, Chunli Wei, Xiaoyan Liu, Zhiqiang Mei, Jingliang Cheng, Kai Wang, Junjiang Fu

**Affiliations:** Key Laboratory of Epigenetics and Oncology, the Research Center for Preclinical Medicine, Southwest Medical University, Luzhou 646000, China

**Keywords:** ADAM17, cancer, SARS-CoV-2, cordycepin (CD), thymoquinone (TQ), N6, N6-Dimethyladenosine (m^6^_2_A), network-pharmacological analysis

## Abstract

COVID-19 is an acute respiratory disease caused by SARS-CoV-2 that has spawned a worldwide pandemic. ADAM17 is a sheddase associated with the modulation of the receptor ACE2 of SARS-CoV-2. Studies have revealed that malignant phenotypes of several cancer types are closely relevant to highly expressed ADAM17. However, ADAM17 regulation in SARS-CoV-2 invasion and its role on small molecules are unclear. Here, we evaluated the ADAM17 inhibitory effects of cordycepin (CD), thymoquinone (TQ), and N6, N6-dimethyladenosine (m^6^_2_A), on cancer cells and predicted the anti-COVID-19 potential of the three compounds and their underlying signaling pathways by network pharmacology. It was found that CD, TQ, and m^6^_2_A repressed the ADAM17 expression upon different cancer cells remarkably. Moreover, CD inhibited GFP-positive syncytia formation significantly, suggesting its potential against SARS-CoV-2. Pharmacological analysis by constructing CD-, TQ-, and m^6^_2_A-based drug-target COVID-19 networks further indicated that ADAM17 is a potential target for anti-COVID-19 therapy with these compounds, and the mechanism might be relevant to viral infection and transmembrane receptors-mediated signal transduction. These findings imply that ADAM17 is of potentially medical significance for cancer patients infected with SARS-CoV-2, which provides potential new targets and insights for developing innovative drugs against COVID-19.

## 1. Introduction

Since December 2019, SARS-CoV-2 has caused the global spread of COVID-19 (Corona Virus Disease 2019), which seriously affects people’s healthy lives [1]. SARS-CoV-2 is an enveloped virus that has caused approximately 300 million infections and more than 5 million deaths worldwide. Multiple receptors that may be associated with SARS-CoV-2 have been identified, such as transmembrane serine protease 2 (TMPRSS2), transmembrane serine protease 4 (TMPRSS4), angiotensin-converting enzyme 2 (ACE2), aminopeptidase N (APN), cathepsin L (CTSL), heparan sulfate proteoglycans (HSPGs), furin, neuropilin-1 (NRP1), heat shock protein A5 (HSPA5), O-acetylated sialic acid (O-Ac-Sia), etc. [2,3,4,5,6,7]. Studies have shown that ACE2 is expressed broadly on the membrane of epithelial cells in the apical region, and especially on the cilium [8]. Coronavirus host cell entry is attributed to ACE2 receptors on the alveolar surface [9,10]. Specifically, the S protein is a very important surface protein of SARS-CoV-2 and exists as a trimer with more than 300 amino acids in each monomer forming a receptor-binding domain that binds tightly to ACE2 and facilitates viral entry of lung cells [11]. ACE2 was found to potentiate cell invasion by the COVID-19-spike (S) protein spseudovirus, but no similar phenomenon was observed in ACE2 mutant cells [12]. Therefore, it seems that cells/tissues expressing ACE2 are susceptible to invasion by this virus. Conversely, in ACE2 mutant mouse models, peak coronavirus injection aggravated acute lung injury and even lung failure, whereas recombinant ACE2 treatment dramatically attenuates acute lung injury [13,14]. On this basis, the researchers proposed the dubious hypothesis that an increase in plasma membrane-bound ACE2 may lead to higher infection rates, although the critical function of ACE2 within COVID-19 infection remains unknown.

A disintegrin and metalloproteinase 17 (ADAM17; OMIM: 603639), also known as TNFα converting enzyme (TACE), CD156B, NISBD1, and CSVP are mainly present in two forms: precursor and activated ADAM17 [15]. ADAM17 facilitates EGF receptor-, TNF-α receptor-, and IL-6 receptor-mediated signal transduction by cleavage/hydrolysis of the ectodomains of more than 80 membrane proteins (e.g., pro-inflammatory cytokines, adhesion molecules, receptors, and growth factors) and regulates multiple pathophysiological processes, including tumor, inflammation, immunity, growth, and metastasis [16,17,18]. Furthermore, ADAM17 activity is also regulated by posttranslational modifications, pro-domain removal, conformational changes, and glycosylation [19]. The proteins iRhom1 and iRhom2 are required for ADAM17 trafficking and enzyme activation [20,21]. ADAM17 is a newly identified SARS-CoV-2-related protein. It is highly expressed in nasopharyngeal, bronchial, and lung tissues [22]. As a type I transmembrane protein and sheddase, ADAM17 probably contributes to the ectodomain shedding of ACE2 [23], leading to the formation of soluble ACE2 and the induction of SARS-CoV-2 virus fusion with host cells [24,25]. Evidence for ADAM17-mediated infection of SARS-CoV-2 in human lung cells suggests that ADAM17 triggers SARS-CoV-2 invasion by cleaving the S protein of the virus [26]. Suppression of ADAM17 by reducing the shedding of ACE2 may reduce viral load and protect host cells from viral infection [27]. In addition to affecting membrane protein shedding, ADAM17 also induces local tumor metastasis and invasion through degradation of the cellular basement membrane and extracellular matrix. The fact that ADAM17 has been reported to be expressed in diverse malignancies, but minimally in normal cells [28], implies its role in tumors. In this regard, ADAM17 inhibition might alleviate the severe infections that occur in patients with malignant tumors. Previously, it was found that ADAM17 expression was higher in tumors than in normal tissues, and that it reduced the prognosis of patients with malignant tumors [22]. Interestingly, ADAM17 was also associated with immune cell infiltration and immunomodulators in tumor and paired normal tissues [22]. These results implicate the importance of ADAM17 in COVID-19-infected cancer patients and provide a new thread for developing anti-COVID-19 drugs.

Chinese herbal medicines and their active ingredients are characterized by multi-targets, multi-pathways, and low toxicity, and are widely used in antiviral and anti-tumor therapy. Cordycepin (CD), N6, N6-dimethyladenosine (m^6^_2_A), and thymoquinone (TQ) are nucleoside analogues with a wide range of pharmacological functions. CD is a natural ingredient originally found in traditional Chinese medicine (TCM) as a medicinal mushroom [29,30]. Our previous studies showed that CD inhibited the malignant progression of drug-resistant non-small cell lung cancer through regulation of the AMPK signaling pathway [29] and repressed triple-negative breast cancer (TNBC) cell migration and invasion by the down regulation of epithelial-to-mesenchymal transition inducible transcription factors (EMT-TFs) [31]. TQ is a biologically active phytochemical component in the seed oil of Panicola nigra [32]. Numerous studies have shown that TQ can protect against cardiovascular diseases [33] and viral infections [34]. Our results suggested that TQ or its analogs synergistically enhanced the antitumor effects of chemotherapeutic agents [35] and repressed the metastasis and invasion of distinct tumor cells [36,37]. m^6^_2_A is a modified ribonucleoside in rRNA and an endogenous A3 adenosine receptor ligand [38,39]. It is also a marine-derived AKT inhibitor [40], and its analogues showed enhanced antitumor effects [41]. CD, TQ, and m^6^_2_A showed anticancer roles both in cells and animals. However, the regulatory effects of CD, TQ, and m^6^_2_A on ADAM17 expression and their potential anti-COVID-19 mechanisms remain unclear.

In this study, we evaluated the ADAM17 inhibitory effects of CD, TQ, and m^6^_2_A on cancer cells and constructed a visual drug-target-disease network to predict prospective targets and mechanisms of the anti-COVID-19 drugs using network pharmacology. 

## 2. Results

### 2.1. The Mutation and Methylation of ADAM17 in Pan-Cancer

**Figure 1:**
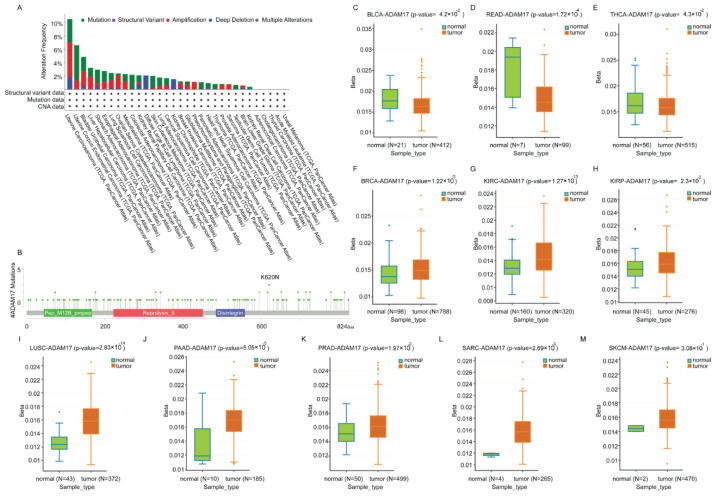
The mutation and methylation of ADAM17 in pan-cancer. (**A**) Summary of ADAM17 mutations across pan-cancerous tissues. Distinct colors show differing mutation types. (**B**) The hot spots of ADAM17 mutations across pan-cancer tissues. (**C**) The methylation of ADAM17 in BLCA. (**D**) The methylation of ADAM17 in READ. (**E**) The methylation of ADAM17 in THCA. (**F**) The methylation of ADAM17 in BRCA. (**G**) The methylation of ADAM17 in KIRC. (**H**). The methylation of ADAM17 in KIRP. (**I**) The methylation of ADAM17 in LUSC. (**J**) The methylation of ADAM17 in PAAD. (**K**) The methylation of ADAM17 in PRAD. (**L**) The methylation of ADAM17 in SARC. (**M**) The methylation of ADAM17 in SKCM. See Figure 2 for the full name of the cancer types. The *p* < 0.05 was considered with statistical significance.

Previously, we studied the expression of ADAM17 in cancer patients and/or normal subjects using bioinformatics analysis. Hereby, we further explored epigenetic modification and gene mutation and their impact on prognosis. Oncogenic mutations may lead to malignant phenotypes of tumors, recurrence, and drug resistance. Through the analysis of mutations in 32 types of cancers, we revealed that ADAM17 expression exhibited the highest mutation frequency, accounting for 10.53% of 57 cases of Uterine Carcinosarcomas, followed by Uterine Corpus Endometrial Carcinoma, accounting for 6.62% of 529 cases, whereas Kidney Renal Clear Cell Carcinoma had the lowest mutation rate, with 0.39% of 511 cases (Figure 1A). ADAM17 mutations were not observed in Adrenocortical Carcinoma, Thyroid Carcinoma, Acute Myeloid Leukemia, Cholangiocarcinoma, and Uveal Melanoma. The detailed landscape of ADAM17 mutations appears to be distributed across the whole AMAD17 region, with missense being the dominant mutation type. We further explored the survival rates, but there was no significant difference between the two groups, although the median monthly survivals (overall, progression-free, disease-specific, and disease free) were longer in the altered group than in the unchanged group (Data not shown).

DNA methylation regulates gene expression. The methylation profiles of 23 types of cancers were analyzed by the DNMIVD database. We found that the methylation status of the ADAM17 promoter in BLCA, THCA, and READ tumor tissues was significantly lower than correlated normal tissues (Figure 1C–E). The methylation status of the ADAM17 promoter was markedly higher in BRCA, KIRC, KIRP, LUSC, PAAD, PRAD, SARC, and SKCM tumor tissues than in paired normal tissues (Figure 1F–M). The hypomethylation of ADAM17 in PAAD tumor tissues appeared to be positively correlated with the higher expression shown in our previous report, implying that hypomethylation of the ADAM17 promotor may be responsible for its increased expression in PAAD tissues. However, promoter methylation in other cancer types may not be the only mechanism regulating ADAM17 overexpression.

### 2.2. Treatment with m^6^_2_A, TQ, and CD Represses ADAM17 Expression in Distinct Cancer Cells

**Figure 2:**
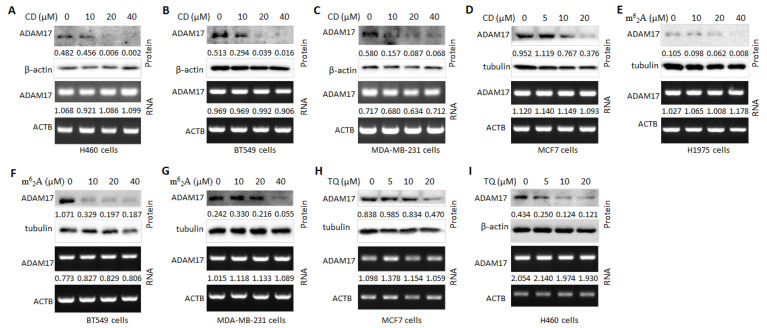
CD, TQ, and m^6^_2_A inhibit ADAM17 expression in distinct cancer cells. (**A**) CD-treated lung cancer cell line H460 was measured for alterations in ADAM17 protein (upper panel) and mRNA (lower panel) levels. (**B**) CD-treated breast cancer cell line BT549 was measured for alterations in ADAM17 protein (upper panel) and mRNA (lower panel) levels. (**C**) CD-treated breast cancer cell line MDA-MB-231 was measured for alterations in ADAM17 protein (upper panel) and mRNA (lower panel) levels. (**D**) CD-treated breast cancer cell line MCF7 was measured for alterations in ADAM17 protein (upper panel) and mRNA (lower panel) levels. (**E**) m^6^_2_A-treated lung cancer cell line H1975 was measured for alterations in ADAM17 protein (upper panel) and mRNA (lower panel) levels. (**F**) m^6^_2_A-treated breast cancer cell line BT549 was measured for alterations in ADAM17 protein (upper panel) and mRNA (lower panel) levels. (**G**) m^6^_2_A-treated breast cancer cell line MDA-MB-231 was measured for alterations in ADAM17 protein (upper panel) and mRNA (lower panel) levels. (**H**) TQ-treated breast cancer cell line MCF7 was measured for alterations in ADAM17 protein (upper panel) and mRNA (lower panel) levels. (**I**) TQ-treated lung cancer cell line H460 was measured for alterations in ADAM17 protein (upper panel) and mRNA (lower panel) levels.

CD, m^6^_2_A, and TQ are nucleoside analogues that have broad pharmacological effects, such as anti-tumor, anti-virus, etc. In this regard, we analyzed the effects of CD, m^6^_2_A, and TQ on ADAM17 levels in different cancer cells. Interestingly, CD, m^6^_2_A, and TQ dose-dependently reduced ADAM17 protein levels in different cancer cells (Figure 2A–I, upper panels), but mRNA levels were not reduced (Figure 2A–I, bottom panels), implying the potential of the three compounds in improving viral susceptibility in patients with malignant tumors.

### 2.3. Treatment with m^6^_2_A or CD Inhibits ADAM17 Translation but Prevents Its Degradation

**Figure 3:**
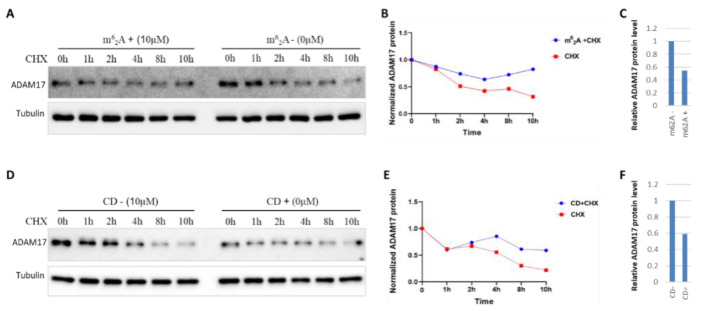
Treatment with m^6^_2_A or CD inhibits the translation, but prevents the degradation of ADAM17 in BT549 cancer cells. (**A**) Stability of the ADAM17 protein was detected in CHX-treated cells in the presence or absence of m^6^_2_A. (**B**) The quantitative results of A. (**C**) Quantitated the ADAM17 protein levels with m^6^_2_A, but without CHX treatment. (**D**) Stability of the ADAM17 protein was detected in CHX-treated cells in the presence or absence of CD. (**E**) The quantitative results of (**D**). (**F**) Quantitated the ADAM17 protein levels with CD, but without CHX treatment. 40 µg/mL was used for the final concentration of CHX.

The stability of the ADAM17 protein was detected in CHX-treated BT549 cells in the presence/ absence of m^6^_2_A. In CHX-treated cells, ADAM17 protein had a half-life of less than 2.3 h, whereas the addition of m^6^_2_A significantly extended the half-life of ADAM17 protein to 4 h (Figure 3A,B). Further, quantitative analysis showed that the combination of m^6^_2_A and CHX markedly decreased the degradation rate of ADAM17 protein by nearly 50% compared to CHX treatment alone (Figure 3C). Such results were then observed in cells treated with CD or CHX alone and their combination (Figure 3D–F). Overall, these results indicate that m^6^_2_A or CD treatment inhibits ADAM17 translation, but prevents its degradation.

### 2.4. Network Pharmacology Analyzes Potential Targets of m^6^_2_A, TQ, and CD

**Figure 4:**
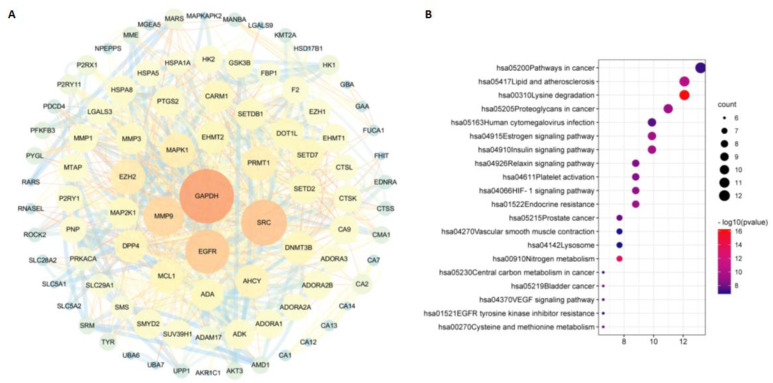
The “m^6^_2_A −targets” network built by Cytoscape 3.6.1. PPI network (**A**) and KEGG pathway enrichment analysis (**B**) of m^6^_2_A. The nodes represent proteins, and the edges refer to interactions between proteins. The size of the circles and their distinct colors represent degrees.

**Figure 5:**
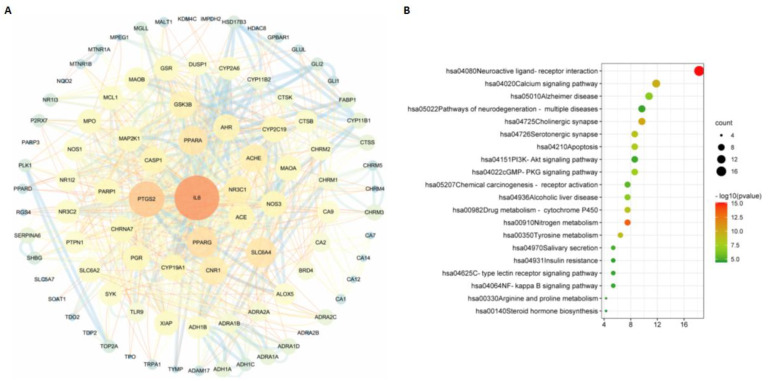
The “TQ−targets” network built by Cytoscape 3.6.1. PPI network (**A**) and KEGG pathway enrichment analysis (**B**) of TQ. The nodes represent proteins, and the edges refer to interactions between proteins. The size of the circles and their distinct colors represent degrees.

**Figure 6:**
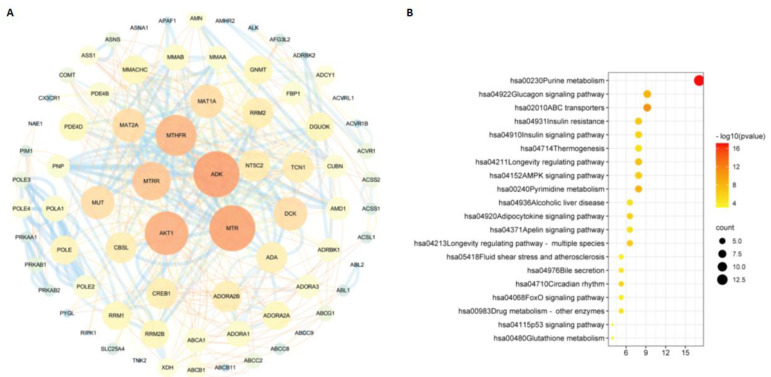
The “CD−targets” network built by Cytoscape 3.6.1. PPI network (**A**) and KEGG pathway enrichment analysis (**B**) of CD. The nodes represent proteins, and the edges refer to interactions between proteins. The size of the circles and their distinct colors represent degrees.

Based on the databases, we collected a total of 100, 100, and 82 potential targets for m^6^_2_A, TQ, and CD, respectively. The interactions of these targets for different compounds analyzed in the STRING database were visualized with Cytoscape software (version 3.6.1). In Figure 4A, Figure 5A, and Figure 6A, we found that ADAM17 is a potential target for m^6^_2_A and TQ, but not CD. To further explore the relationship between the three compounds and their targets, KEGG analysis was performed. A total of 134 pathways of m^6^_2_A, 75 pathways of TQ, and 71 pathways of CD were enriched by KEGG analysis (adjusted *p* < 0.01). Based on the GeneRatio values and p-value, the top 20 pathways of these compounds were selected, e.g., pathways in cancer, human cytomegalovirus infection, lipid and atherosclerosis, chemical carcinogenesis-receptor activation, neuroactive ligand-receptor interaction, purine metabolism, calcium signaling pathway, glucagon signaling pathway, and ABC transporters, and were shown in Figure 4B, Figure 5B, and Figure 6B.

### 2.5. Construction of the Compounds-Targets-COVID-19 Network

**Figure 7:**
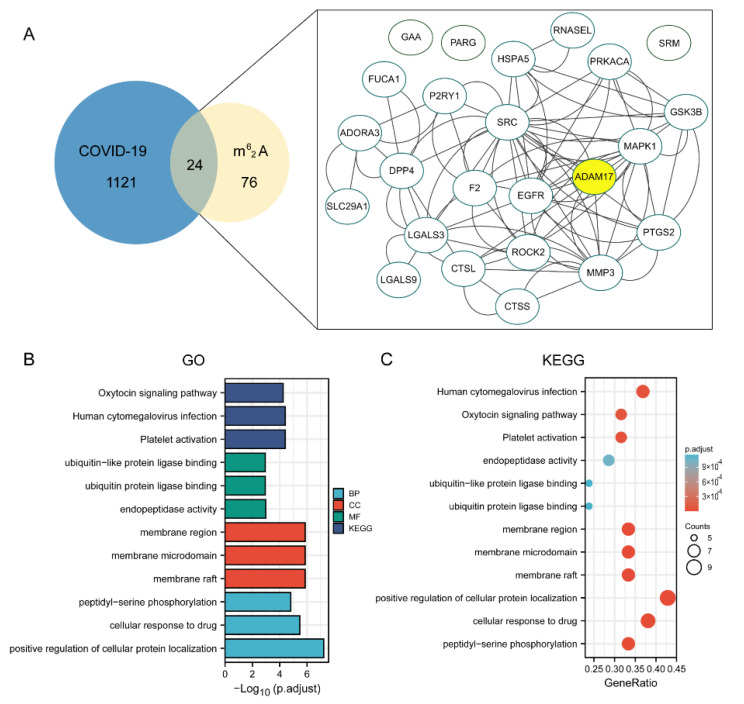
Establishment of an ingredient-target-disease network for m^6^_2_A in COVID-19. (**A**) The Venn diagram exhibited a total of 24 m^6^_2_A targets for COVID-19 were extracted. ADAM17 was one of the key targets. Enrichment analysis of pathways for the 24 hub genes. (**B**) The top 3 terms of BP, CC, and MF enrichment. (**C**) Enrichment analysis of pathways for the 24 DEGs.

**Figure 8:**
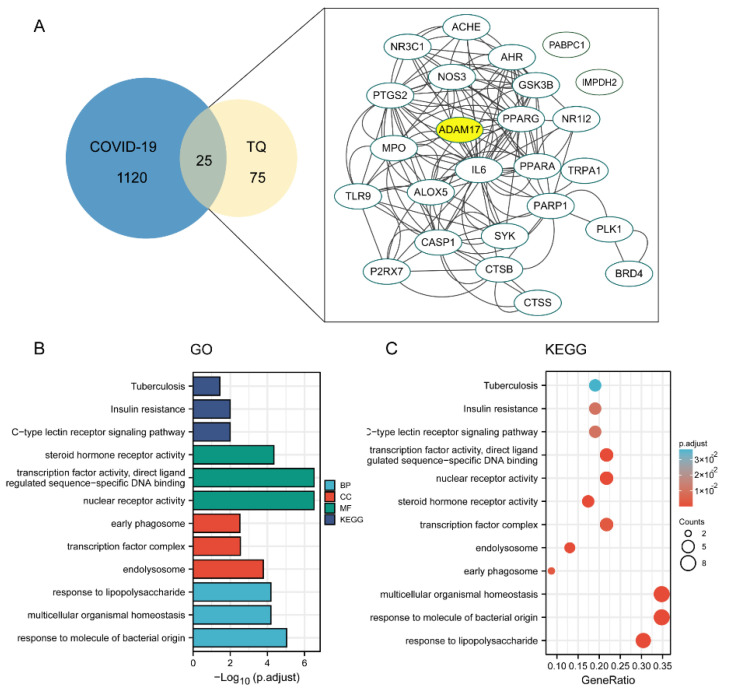
Establishment of an ingredient-target-disease network for TQ in COVID-19. (**A**) The Venn diagram exhibited a total of 25 TQ targets for COVID-19 were extracted. ADAM17 was one of the key targets. Enrichment analysis of pathways for the 25 hub genes. (**B**) The top 3 terms of BP, CC, and MF enrichment. (**C**) Enrichment analysis of pathways for the 25 DEGs.

**Figure 9:**
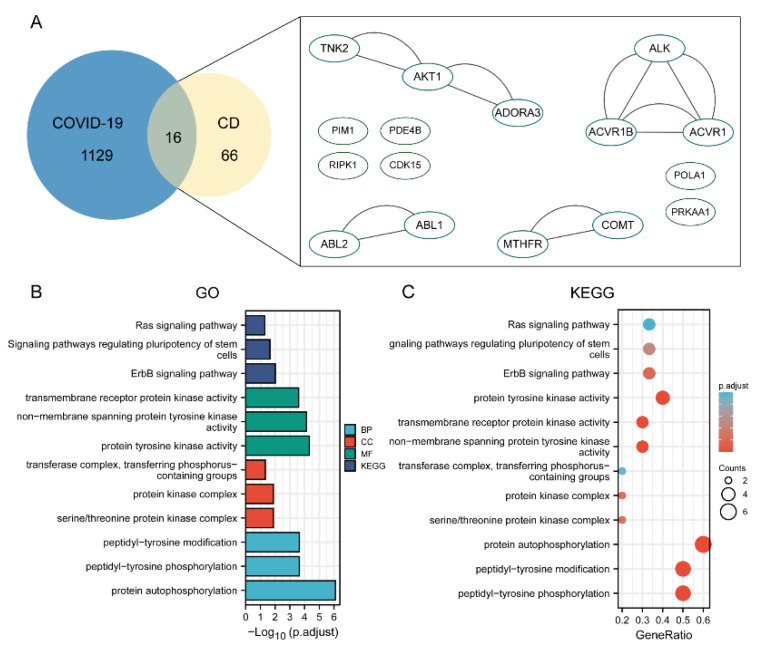
Establishment of an ingredient-target-disease network for CD in COVID-19. (**A**). The Venn diagram exhibited a total of 16 CD targets for COVID-19 were extracted. Enrichment analysis of pathways for the 16 hub genes. (**B**) The top 3 terms of BP, CC, and MF enrichment. (**C**) Enrichment analysis of pathways for the 16 DEGs.

To elucidate the mechanism of the compounds against COVID-19, we collected the common differentially expressed genes of the three constructed compound-target networks and 1145 COVID-19-associated target gene networks retrieved from the databases. Protein-protein interactions were obtained by uploading the above described candidate targets to the STRING database and assigning p-values less than 1, 10, and 16. We found that ADAM17 is also one of the larger nodes, indicating that ADAM17 may play a very important role in the anti-COVID-19 effect of the three compounds (Figure 7A, Figure 8A, and Figure 9A). We further conducted GO enrichment/KEGG analysis to investigate the potential mechanism of the three compounds in COVID-19, using the DAVID database. 

For GO-term analysis, key terms of BP enrichment include: (1) m^6^_2_A: peptidyl-serine phosphorylation, cellular response to the drug, positive regulation of cellular protein localization; (2) TQ: response to lipopolysaccharide, multicellular organismal homeostasis, response to molecule of bacterial origin; (3) CD: peptidyl-tyrosine modification, protein autophosphorylation, peptidyl-tyrosine phosphorylation (Figure 7B, Figure 8B, and Figure 9B). MF analysis was mainly enriched in (1) m^6^_2_A: ubiquitin-like protein ligase binding, endopeptidase activity, ubiquitin protein ligase binding; (2) TQ: steroid hormone receptor activity, transcription factor activity, nuclear receptor activity, direct ligand regulated sequence-specific DNA binding; (3) CD: transmembrane receptor protein kinase activity, non-membrane spanning protein tyrosine kinase activity, protein tyrosine kinase activity (Figure 7B, Figure 8B, and Figure 9B). CC terms revealed that: (1) m^6^_2_A was associated with membrane region, membrane microdomain, and membrane raft; (2) TQ was associated with the early phagosome, transcription factor complex, and endolysosome; (3) CD was associated with transferring phosphorus-containing groups, transferase complex, protein kinase complex, and serine/threonine protein kinase complex (Figure 7B, Figure 8B, and Figure 9B). For KEGG analysis, multiple candidate genes have interacted with COVID-19-related pathways. Among them, virus infection and protein phosphorylation were considered 2 of the top 12 enrichment pathways associated with these compounds (Figure 7C, Figure 8C, and Figure 9C). Additionally, the anti-COVID-19 mechanism may also be related to ubiquitin-like protein ligase binding, membrane microdomain, membrane raft, direct ligand-regulated sequence-specific DNA binding, endolysosome, and transmembrane receptor protein kinase activity.

### 2.6. CD Is Able to Inhibit Syncytia Formation

Emerging evidence indicates that a large number of multinucleated cells characteristic of syncytial pathology are present in patients with COVID-19 [42], which is a pathological hallmark of SARS-CoV-2 infection. Syncytium formation is required for the participation of the SARS-CoV-2 Spike protein and the host cell ACE2 [26]. Here, SARS-CoV-2-Spike plasmids with GFP fluorescence were transfected into 293T-ACE2 cells and co-incubated with untransfected 293T-ACE2 cells for 24 h, followed by the addition of 20 μM CD for another 24 h. We observed many larger syncytia with GFP green fluorescence in control cells, indicating SARS-CoV-2 cell invasion (Supplementary Figure S1A). However, the area of fluorescence of GFP-positive syncytia was significantly reduced upon treatment with CD (Supplementary Figure S1A). Further, quantitative analysis revealed that the mean fluorescence area of CD-treated syncytia was reduced to 44.34% ± 25.54 compared to the control group (Supplementary Figure S1B).

## 3. Discussions

COVID-19 has become a global public health problem. It is of great help to understand the expression of potential SARS-CoV-2 receptors (e.g., ACE2 and ADAM17) in host cells/tissues to reduce the replication and transmission of the virus and the severity of COVID-19. Studies have shown that ACE2 is involved in the viral invasion of host cells, leading to infection by binding to the B domain of the COVID-19 virus’s S protein [43,44,45]. Similar to ACE2, ADAM17 is another newly identified virus recognition receptor [46], and its distributed and expressed levels may mirror the susceptibility of the virus, its replication, and its invasion. Inhibition of ADAM17 expression protects the body from COVID-19 infection [47]. Increasing evidence indicates that ADAM17 is expressed in multiple malignancies at higher levels than in paired normal tissues [28,48], suggesting its specificity in tumors. Moreover, invasion and metastasis of distinct malignancies can be attributed to ADAM17-mediated degradation of the cellular basement membrane and extracellular matrix [48]. However, the underlying role of ADAM17 in cancer patients infected with COVID-19 remains unknown.

In a previously published paper [22], we explored ADAM17 protein or its mRNA expression in pan-cancer and adjacent normal tissues. It was found that ADAM17 was significantly higher in multiple human tumor tissues than in adjacent tissues, and that cancer patients with high ADAM17 expression had a poor clinical prognosis. Gene mutations favor the progression, recurrence, and chemo-resistance of malignant tumors, and DNA methylation can provoke structural changes in chromosomes that lead to tumorigenesis by turning off tumor suppressor genes. Actually, point mutations of the ADAM17 catalytic domain have been identified in tumor samples from cancer patients, which are associated with tumor-related dysfunction [49]. Meanwhile, protein posttranslational modifications have been found to be closely relevant to the regulation of ADMA17 activation [50,51]. We speculate that both may probably be related to tumor progression. In this study, ADAM17 was found to be expressed in 57 cases of uterine carcinosarcoma with the highest mutation frequency (10.53%), while it was barely expressed in 511 cases of kidney renal clear cell carcinoma with a mutation rate of 0.39%. By analyzing the methylation profiles, the methylation status of the ADAM17 promoter in BLCA, READ, and THCA tumor tissues was distinctly lower than in adjacent normal tissues, whereas its methylation status was higher in BRCA, KIRC, KIRP, LUSC, PAAD, PRAD, SARC, and SKCM tumor tissues. Our previous study indicated that hypomethylation of ADAM17 in PAAD tumor tissues was positively correlated with high ADAM17 expression, indicating that hypomethylation of the ADAM17 promoter may be responsible for its increased expression in PAAD tissues. However, promoter methylation in other tumor types may not be the only mechanism regulating ADAM17 overexpression. In addition, the catalytic domain of ADAM17 is required for the cleavage of the substrates [50]. The cleavage and release of some substrates, including ACE2, directly drive viral invasion [11]. Hence, it is not difficult to speculate about the effect of site-specific mutations or post-translational modifications of the catalytic domain on the shedding activity of ADAM17 and viral invasion.

Alterations in ADAM17 expression affect susceptibility to viral infection and the severity of COVID-19, implying the importance of targeting ADAM17 in patients with malignant tumors infected with COVID-19 [47]. CD is a nucleoside derivative extracted from Cordyceps Sinensis with a wide-range of biological activities, including antiviral replication, anticancer, anti-inflammatory, antidepressant, hepatic, neuroprotective, etc. [29,52,53]. Recent studies have shown that CD is able to conjugate to the S protein and Mpro protein of SARS-CoV-2. In vitro SARS-CoV-2 invasion assay supports the antiviral effect of CD [54]. In this study, CD suppressed ADAM17 expression, especially in lung cancer, which supports the possibility of developing anti-SARS-CoV-2 drugs and suggests a role for CD in anti-SARS-CoV-2 therapy in cancer patients by inhibiting ADAM17 expression. m^6^_2_A, another nucleoside derivative, exhibited an inhibitory effect on CTSL protein expression. TQ, a major component of N. Sativa, has been reported to potentially suppress the development of COVID-19 by binding to TMPRSS2 [32,55]. TQ exerts a suppressive effect on the malignant progression of cancer cells [36,56]. Chase assays confirmed that treatment with m^6^_2_A or CD increased the stability of ADAM17 protein, but significantly decreased its total protein levels, suggesting that m^6^_2_A/CD treatment alone inhibits ADAM17 translation, but prevented its degradation. The different mechanisms may be connected with the action of m^6^_2_A. Collectively, our results showed that CD, m^6^_2_A, and TQ remarkably reduced ADAM17 protein levels in distinct tumor cells. A large number of multinucleated cells characteristic of syncytial pathology are present in COVID-19 patients [42], which is a pathological hallmark of SARS-CoV-2 infection. Syncytium formation is required for the participation of SARS-CoV-2 Spike protein and ACE2 protein of the host cells [26]. Our results showed many large syncytia with GFP green fluorescence in control cells, whereas the area of fluorescence of GFP-positive syncytia was significantly reduced upon addition of CD. The above results suggest a potential for these drugs against SARS-CoV-2, although further studies are needed.

Network pharmacology is an approach based on systematic biological theory to predict underlying molecular mechanisms through high-throughput virtual computing and database retrieval, as well as the establishment of the drug (compound)-target-disease-pathway network. Recently, a large number of studies have employed this approach to explain the complex interrelationships between drugs and diseases. Herein, the relationship between anti-COVID-19 treatment and m^6^_2_A/TQ/CD treatment was fully explored by network pharmacology. These data suggested that 100, 100, and 82 targets were respectively associated with m^6^_2_A, TQ, and CD on COVID-19, in which (GAPDH, MMP9, SRC, EGFR, EZH2, MAPK1, PRMT1), (IL-6, PTGS2, PPARG, SLC6A4, PPARA, ACHE, NR3C1), and (ADK, MTR, AKT1, MTHFR, MTRR, MUT, MAT1A, DCK) were considered as core targets, respectively. It has been reported that SRC [57], EGFR [58], EZH2, IL-6 [59], PTGS2 [60], PPARG [61], SLC6A4 [62], PPARA [63], ACHE [64], NR3C1 [65], ADK [66], AKT1 [67], and MTHFR [68] are closely related to the pathogenesis of COVID-19. Interestingly, we found that ADAM17 is a potential target for m^6^_2_A and TQ, rather than CD, which may be related to the number of articles published in these years. Further, 134 m^6^_2_A-related pathways, 75 TQ-related pathways, and 71 CD-related pathways were enriched by KEGG enrichment analysis, among which 20 pathways were significantly enriched, including pathways in cancer, human cytomegalovirus infection, lipid and atherosclerosis, chemical carcinogenesis-receptor activation, purine metabolism, calcium signaling pathway, neuroactive ligand-receptor interaction, glucagon signaling pathway, ABC transporters, etc. In recent years, evidence has shown that COVID-19 is implicated in neuroactive ligand-receptor interactions [69], and calcium signaling pathways conduce to the shedding of ACE2 catalytic outer domains during COVID-19 infection [70]. Purine metabolism is found to be associated with COVID-19 infection [71]. According to the findings of the network pharmacology analysis, we identified the regulatory mechanisms of m^6^_2_A, TQ, and CD in COVID-19 disease involving multiple pathways and multiple targets. Protein-protein interaction results indicated that ADAM17 might play critical roles in the anti-COVID-19 activity of these three compounds. Further studies revealed that multiple candidate genes interact with COVID-19-related pathways, with the viral infection and protein phosphorylation identified as 2 of the top 12 enrichment pathways associated with these compounds. In addition, sequence-specific DNA-binding endolysosome and transmembrane receptor protein kinase activity directly regulated by ligands in the ubiquitin-like protein ligase membrane microdomain may also be the mechanism of its resistance to COVID-19. Possible explanations for the mechanism may be as follows: ADAM17 is involved to some extent in the potential antiviral effects of small molecule compounds of traditional Chinese medicine. ADAM17 also is a shedding enzyme that helps shed and activate nearly 100 substrates and, thus, possesses a wide range of biological effects. Some core targets (such as EGFR, IL-6, ACE2, etc.) in the “drug-target” network are the direct substrates of ADAM17. Although it does not play a primary role in the network, ADAM17 probably mediates the shedding and activation of substrates EGFR [72], IL-6 [73,74,75], and ACE2 [76] and then causes inflammation and immune responses, which further demonstrates the multi-target and versatility of the active molecules of traditional Chinese medicine.

## 4. Materials and Methods

### 4.1. Online Databases

ADAM17 promoter DNA methylation analysis was conducted with the DNA Methylation Interactive Visualization Database (DNMIVD) [77]. The ADAM17 mutation was analyzed by the cBioPortal for Cancer Genomics [4,78].

### 4.2. Cell Culture and Drug Treatments

Human lung adenocarcinoma cells (H1975 and H460) and breast cancer cells (BT549, MDA-MB-231, and MCF7) were supplied by ATCC (American Type Culture Collection) (VA, USA). BT549 cells and H1975 were cultured in RPMI1640 medium containing 10% FBS (fetal bovine serum) and 1% penicillin/streptomycin (PS). The remaining 3 cells were maintained in DMEM mediums with 10% FBS and 1% PS. All the cells were incubated at 37 °C in a 5% CO_2_ incubator. For the following in vitro experiments, cells were seeded into 12-well plates and treated with distinct concentrations of cordycepin (CD, 10–40 μM), thymoquinone (TQ, 5–20 μM), and N6, N6-Dimethyladenosine (m^6^_2_A, 10–40 μM) for 24 h. The whole protein lysates were gathered and analyzed by Western blotting. Total RNA was extracted and reverse transcription was conducted.

### 4.3. Western Blotting and Chase Assays

Western blotting was performed as described previously [79]. The PVDF membranes with quantified protein samples were blocked with 5% non-fat milk and incubated with a primary antibody to ADAM17 (cat #: HPA051575, Sigma-Aldrich), β-actin, or tubulin in 2% fat-free milk at 4 °C and blotted with a secondary antibody at ambient temperature. Protein bands were exposed and photographed using the Syngene G: BOX Imaging System (Cambridge, UK). Chase assays for ADMA17 protein stability were performed with indicated cycloheximide (CHX) and m^6^_2_A or CD in BT549 cancer cells.

### 4.4. Semi-Quantitative RT-PCR

For the detection of ADAM17 mRNA, the total RNA of different tumor cells was extracted, as per instructions from the manufacturer, using a Total RNA Extraction Kit. Synthesized cDNAs were subjected to semi-quantitative RT-PCR with forward primer 5′-cccaccagagactcgagaag-3′ and the reverse primer 5′-caaccacgtgtccagtgaag-3′ [80]. The product size is 279 bp. ACTB was used as the internal control.

### 4.5. Establishment of Small Molecule Compounds-Target Interaction

TQ, m^6^_2_A, and CD were searched for in the Traditional Chinese Medicine Systems Pharmacology (TCMSP, http://tcmspw.com/tcmsp.php, accessed on 15 January 2022), Herbal Ingredients’ Targets Database (HIT, http://lifecenter.biosino.org/hit/, accessed on 15 January 2022), Traditional Chinese medicine integrative database for herb molecular mechanism analysis (TCMID, http://www.megabionet.org/tcmid/, accessed on 15 January 2022), TCM@Taiwan (http://tcm.cmu.edu.tw, accessed on 15 January 2022), CancerHSP (http://ibts.hkbu.edu.hk/LSP/CancerHSP.php, accessed on 15 January 2022), Naturally occurring Plant based Anticancerous Compound-Activity-Target DataBase (NPACT, http://crdd.osdd.net/raghava/npact/, accessed on 15 January 2022), Natural Product Activity and Species Source Database (NPASS, http://bidd2.nus.edu.sg/NPASS, accessed on 15 January 2022) system network-pharmacological databases. Drug likeness (DL) of greater than 0.18 and oral bioavailability (OB) of greater than 30% were employed as criteria for screening potential compounds. Cytoscape software (version 3.6.1) for the construction and visualization of the interactions between the three compounds and the target was used.

### 4.6. Gene Ontology and Pathway Enrichment Analysis

COVID-19-related gene expression data were obtained from the GeneCards^®^: The Human Gene Database (https://www.genecards.org/, accessed on 12 September 2021) and Gene Expression Omnibus Database (https://www.ncbi.nlm.nih.gov/geo/, accessed on 12 September 2021). The genes that correlate with SARS-CoV-2 infection and negative control in the gene expression profile of this dataset were analyzed using the GEN2R online tool. A total of 1145 differentially expressed genes (DEGs) were obtained based on *p* ≤ 0.05 and fold change (FC) ≥ 1.5. Protein-protein interaction (PPI) analyses were performed using the STRING database (https://cn.string-db.org/, accessed on 16 January 2022) to obtain common genes. The Kyoto Encyclopedia of Genes (KEGG) and Gene Ontology (GO) analyses were carried out by the DAVID database (http://david.abcc.ncifcrf.gov/, accessed on 16 January 2022). Go annotations were used to analyze the following three terms: cellular composition (CC), biological processes (BP), and molecular function (MF).

### 4.7. Syncytia Formation Assay

Syncytia formation is the hallmark event in SARS-CoV-2 cellular infections [26]. In this study, SARS-CoV-2-Spike plasmids with GFP fluorescence pCDH-CMV-HnCoV-S-EF1-copGFP purchased from Shanghai HedgehogBio Science and Technology Ltd. were transfected into 293T-ACE2 cells and co-incubated with untransfected 293T-ACE2 cells for 24 h, followed by the addition of 20 μM CD for another 24 h. Syncytia formation in each group was observed and analyzed using a ZOE Fluorescent Cell Imager (Bio-Rad, Hercules, CA, USA).

## 5. Conclusions

In conclusion, hypomethylation of ADAM17 was positively correlated with high ADAM17 expression in PAAD tumor tissues, suggesting that hypomethylation of the ADAM17 promoter may be responsible for the increased ADAM17 expression in PAAD tissues. In addition, ADAM17 protein levels were significantly reduced in lung and breast cancer cells treated with nucleoside compounds, such as CD, TQ, and m^6^_2_A. CD significantly decreased the area of fluorescence of GFP-positive syncytia compared to the control group. Further, a network pharmacology approach was employed to establish a drug-disease-target network and analyze the potential molecular pathways by GO and KEGG enrichment analysis. Overall, this research not only insinuates the medical significance of ADAM17 for COVID-19 cancer patients, but it also sheds potential light on the treatment of COVID-19.

## Data Availability

Not applicable.

## Supplementary Materials

The following supporting information can be downloaded at: https://www.mdpi.com/article/10.3390/molecules27249044/s1, Figure S1: CD significantly inhibits syncytia formation.

## Abbreviations

SARS-CoV-2	Severe acute respiratory syndrome coronavirus 2
COVID-19	Coronavirus Disease 2019
ADAM17	A disintegrin and metalloproteinase domain 17
ATCC	American Type Culture Collection
CD	Cordycepin
TQ	Thymoquinone
m^6^_2_A	N6, N6-Dimethyladenosine
CHX	Cycloheximide
TCMSP	Traditional Chinese Medicine Systems Pharmacology
TCMID	Traditional Chinese medicine integrative database
HIT	Herbal Ingredients’ Targets Database
NPACT	Naturally occurring Plant based Anticancerous Compound-Activity-Target DataBase
NPASS	Natural Product Activity and Species Source Database
DL	Drug likeness
OB	Oral bioavailability
DEGs	Differentially expressed genes
KEGG	Kyoto Encyclopedia of Genes
GO	Gene ontology
PPI	Protein-protein interaction
CC	Cellular composition
BP	Biological processes
MF	Molecular function
