# Supplementary material for: Antiviral Potential of Small Molecules Cordycepin, Thymoquinone, and N6, N6-Dimethyladenosine Targeting SARS-CoV-2 Entry Protein ADAM17
Source: Molecules. 2022 Dec 19;27(24):9044. doi: 10.3390/molecules27249044 (PMC9781528; doi:10.3390/molecules27249044)
Supplement: Supplementary file 1 [file molecules-27-09044-s001.zip › molecules-2028431-supplementary.pdf]

# Supplementary Figure

## Supplementary Figure S1

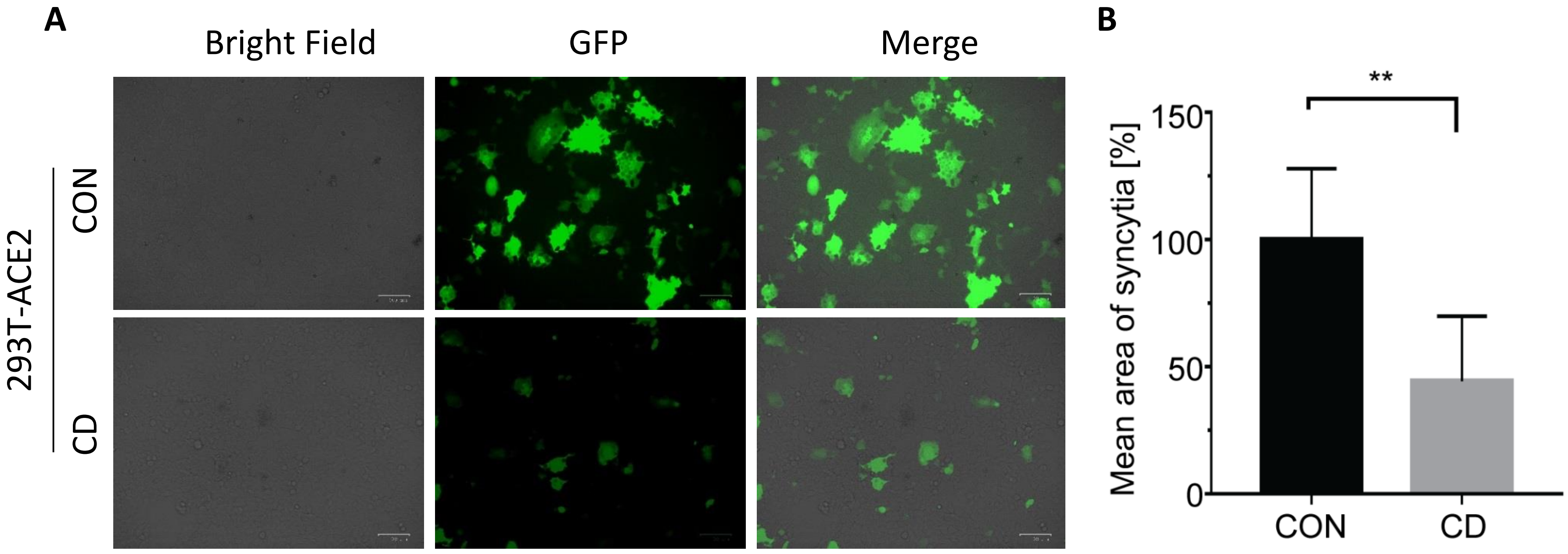


**Supplementary Figure S1. CD significantly inhibits syncytia formation.** A. Representative images of syncytia formation in control and CD-treated 293T-ACE2 cells. B. The quantitative
